# Programs and Place: Risk and Asset Mapping for Fall Prevention

**DOI:** 10.3389/fpubh.2017.00028

**Published:** 2017-03-16

**Authors:** Matthew Lee Smith, Samuel D. Towne, Audry S. Motlagh, Donald R. Smith, Ali Boolani, Scott A. Horel, Marcia G. Ory

**Affiliations:** ^1^Department of Health Promotion and Behavior, College of Public Health, Institute of Gerontology, The University of Georgia, Athens, GA, USA; ^2^Department of Health Promotion and Community Health Sciences, School of Public Health, Texas A&M University, College Station, TX, USA; ^3^Johns Hopkins Bayview Medical Center, Community Psychiatry Program, Baltimore, MD, USA; ^4^United Way of Tarrant County, Fort Worth, TX, USA; ^5^Clarkson University, Potsdam, NY, USA

**Keywords:** asset mapping, risk assessment, older adults, fall prevention, strategic planning

## Abstract

Identifying ways to measure access, availability, and utilization of health-care services, relative to at-risk areas or populations, is critical in providing practical and actionable information to key stakeholders. This study identified the prevalence and geospatial distribution of fall-related emergency medical services (EMS) calls in relation to the delivery of an evidence-based fall prevention program in Tarrant County, Texas over a 3-year time period. It aims to educate public health professionals and EMS first respondents about the application of geographic information system programs to identify risk-related “hot spots,” service gaps, and community assets to reduce falls among older adults. On average, 96.09 (±108.65) calls were received per ZIP Code (ranging from 0 calls to 386 calls). On average, EMS calls per ZIP Code increased from 30.80 (±34.70) calls in 2009 to 33.75 (±39.58) calls in 2011, which indicate a modest annual call increase over the 3-year study period. The percent of ZIP Codes offering A Matter of Balance/Volunteer Lay Leader Model (AMOB/VLL) workshops increased from 27.3% in 2009 to 34.5% in 2011. On average, AMOB/VLL workshops were offered in ZIP Codes with more fall-related EMS calls over the 3-year study period. Findings suggest that the study community was providing evidence-based fall prevention programming (AMOB/VLL workshops) in higher-risk areas. Opportunities for strategic service expansion were revealed through the identification of fall-related hot spots and asset mapping.

## Introduction

Identifying ways to measure access, availability, and utilization of health-care services, relative to at-risk areas or populations, is critical in providing practical and actionable information to key stakeholders. This is especially important in efforts to ameliorate potentially preventable health-related complications or poor health outcomes among a rapidly aging population of community-dwelling older adults. Several interrelated and health-related issues face older adults, including falls (1), low physical activity levels (2), and chronic disease and related complications (3). However, many preventable health issues may be targeted with evidence-based approaches. Non-clinical approaches or interventions that target risk factors for preventable complications associated with the aging process can include evidence-based health and wellness program delivered in community settings to community-dwelling older adults. One such evidence-based program targets risk of falling and confidence associated with preventing a fall, namely A Matter of Balance in the form of the Volunteer Lay Leader (e.g., non-clinician lead) also known as AMOB/VLL (4–6). The risk of falling is broadly related to both physical activity and chronic disease.

Falls among older adults are a growing public health issue, with one in every four adults aged 65 years falling each year (7). Furthermore, falls prevalence is even greater among those aged 75 years and older, and the odds of repeated falls increase after the fall-related incidence (8). Falls are among the leading cause of preventable death among older adults and are associated with morbidity, functional limitations, loss of independence, and increased direct and indirect health-care costs (1). A large proportion of falls require emergency medical services (EMS) to be dispatched, and falls account for an estimated 15% of all EMS calls in some communities (9, 10). However, of all fall-related EMS calls in the U.S., approximately 21% did not result in transfer/transport to health-care facilities (11).

### Asset Mapping

Asset mapping is a useful tool for assessing health-related needs, disparities, and inequities within communities (12). Ordinarily used to visualize trends in environmental, epidemiological, and analysis of biostatistical data, the use of geographic information systems (GIS) is currently utilized for the organization of social services available in the community to illustrate geographic proximity or distance to its intended targets (13). Visually layering sociodemographic data on top of data showing services offered can reveal a variety of community needs in specific neighborhoods or areas. This nuance in community development, if used properly, can aid in the distribution of grants and funds as well as identify organizations and populations that are in need of assistance (14). Examples of such research includes identifying the reach of evidence-based health and wellness programs targeted to older community-dwelling adults across traditionally low-resource setting (e.g., rurality) (15) and by the density of programs delivered (e.g., delivery of one, two, or more programs in a defined geographic area) (16). While these are examples of national efforts, other studies can target state-based delivery of such evidence-based programs (17). One such study examined state-specific data combining both fall-related hospitalizations to identify hot spots throughout the state relative to evidence-based program delivery (17). While previous findings were focused on hospitalization data, this approach or model of asset mapping can be translated broadly to identify different health-related outcomes (e.g., fall-related emergency medical services or EMS calls in a defined geographic area).

The prevalence of EMS calls in a community is one of many fall-related risk indicators. The geospatial distribution of EMS calls can indicate a higher density of older adult residents in a given area, disproportionate environmental risk, or an absence of fall prevention strategies and solutions to offset risk. As such, tracking EMS calls has the potential to diagnose community-level aliments and enhance strategic planning efforts for fall prevention that involve EMS first responders and other community-based fall prevention interventions.

This study identified the prevalence and geospatial distribution of fall-related EMS calls in relation to the delivery of an evidence-based fall prevention program in Tarrant County, Texas over a 3-year time period. It aims to educate public health professionals and EMS first respondents about the application of GIS programs to identify risk-related “hot spots,” service gaps, and community assets to reduce falls among older adults. We identified an example of integrating different data layers in the form of asset mapping highlighting fall-related EMS calls to relative at-risk areas and populations; with the goal of translating findings to plan and coordinate services to meet deficient needs in community settings. We analyzed the distribution of *risks* (e.g., at-risk areas or at-risk populations) and *assets* (e.g., the availability of AMOB/VLL) within one Texas County (Tarrant County). The primary research questions that guided this study were (a) What was the prevalence of fall-related EMS calls in Tarrant County, Texas over a 3-year period? (b) What was the prevalence of AMOB/VLL delivery in Tarrant County, Texas over a 3-year period? and (c) was there an association between fall-related EMS calls and AMOB/VLL delivery in Tarrant County Zip Codes over a 3-year period? Identifying assets in relation to at-risk areas or populations can provide practical and actionable information that service deliverers and program planners can use to identify gaps and strengthen relationships and collaborative partnerships. The strengths and weaknesses of this risk and asset mapping technique will be discussed in terms of strategic planning for resource/intervention delivery in community settings.

## Materials and Methods

### A Matter of Balance/Volunteer Lay Leader Model

A Matter of Balance/Volunteer Lay Leader Model is an evidence-based fall risk reduction program that utilizes cognitive-behavioral principles of behavior change to reduce the fear of falling and increase physical activity among older adults (18, 19). The program is delivered in a small group format. Each workshop consists of eight interactive sessions, each session lasting for approximately 2 hours. The workshop can be delivered over a 4- or 8-week period (sessions occurring twice or once per week, respectively) (18). Trained volunteer lay leaders facilitate the workshops, each of which have access to a training manual and two instructional videos (20). As described elsewhere, the curriculum includes lectures, group discussions, mutual problem solving, role-play activities, exercise training, assertiveness training, and home assignments (20). The intervention has been shown to be effective to improve participants’ fall-related self-efficacy as well as improve physical and mental health indicators (5, 21–27).

### Tarrant County, Texas

Tarrant County was selected as the area of study because of their long-standing history implementing a variety of fall prevention and disease self-management programs through the aging services network and their membership within the Evidence-Based Leadership Council. The United Way of Tarrant County, located in Fort Worth, Texas, has repeatedly competed successfully for government funding to implement evidence-based programs for older adults and is widely recognized as a community leader and innovator in the evidence-based movement for older adults in the U.S. According to the U.S. Census Bureau, in 2010 Tarrant County spanned 863.61 square miles, with 2,094.7 inhabitants per square mile (28). In 2010, Tarrant County had an estimated total of 1,809,034 residents, which was projected to have grown by 9.6% by 2015 (28). Of the county residents in 2010, 8.9% were aged 65 years and older, 51.0% were female, 26.7% were Hispanic or Latino, 14.9% were Black or African American, and 13.1% were considered to be living in poverty (28).

### Measures

Data utilized for this study were gathered for the years 2009, 2010, and 2011 from three secondary data sources. First, data were requested from the Fort Worth Fire Department (FWFD) about fall-related EMS calls. These data encompassed 44 ZIP Codes in the Fort Worth area. Data obtained from the FWFD included the ZIP Code and geographic coordinates (longitude and latitude) associated with each fall-related EMS call. These data points were plotted using ArcGIS.

Second, AMOB/VLL delivery site locations were obtained from the Tarrant County United Way. The AMOB/VLL workshops were delivered by trained facilitators who were certified by Maine Health. Data obtained from the Tarrant County United Way included the addresses of organizations where AMOB/VLL workshops were delivered. These data encompassed 55 ZIP Codes in Tarrant County (i.e., the 44 Fort Worth ZIP Codes and 11 additional surrounding ZIP Codes). Data points were plotted using ArcGIS.

Third, U.S. Census data were used to determine the proportion of residents that were aged 65 and older in each ZIP Code of interest. ZIP Codes were shaded based on their proportion of older adult residents (darker shading indicates a larger proportion of older adult residents).

### Data Analysis

Data were analyzed using SPSS (version 24). Frequencies and descriptive statistics were calculated for fall-related EMS calls and AMOB/VLL delivery across ZIP Codes. Because of the small number of ZIP Codes included in this study (*n* = 44) and based on the non-normal distribution of our fall-related EMS call data (i.e., presence of substantial outliers), non-parametric analyses (i.e., Kruskal–Wallis tests) were performed. ArcGIS (version 10.2) was used to map geospatial data. A series of maps were generated to examine the distribution of fall-related EMS calls relative to the proportion of residents aged 65 years and older and AMOB/VLL workshop delivery per ZIP Code.

## Results

As seen in Table 1, in the 44 Fort Worth ZIP Codes, a total of 4,228 EMS calls were received from 2009 to 2011. On average, 96.09 (±108.65) calls were received per ZIP Code (ranging from 0 call to 386 calls). The number of EMS calls increased somewhat across years, with 1,355 calls received in 2009, 1,388 calls received in 2010, and 1,485 calls received in 2011. On average, the number of EMS calls per ZIP Code also increased, with 30.80 (±34.70) calls per ZIP Code in 2009 (ranging from 0 to 120 calls), 31.55 (±36.21) calls per ZIP Code in 2010 (ranging from 0 to 124 calls), and 33.75 (±39.58) calls per ZIP Code in 2011 (ranging from 0 to 162 calls).

**Table 1:** **Fall-related EMS calls by A Matter of Balance/Volunteer Lay Leader Model (AMOB/VLL) workshop frequency**.

	ZIP Codes delivered AMOB/VLL between 2009 and 2011						

	Total (*n* = 44)	0 times (*n* = 24)	1–5 times (*n* = 15)	6–10 times (*n* = 5)	Min	Max	Median
2009 EMS fall events	30.80 (±34.70)	23.13 (±26.89)	29.67 (±35.75)	71.00 (±43.93)	0	120	16.50
2010 EMS fall events	31.55 (±36.21)	25.63 (±30.86)	27.67 (±34.63)	71.60 (±46.36)	0	124	17.50
2011 EMS fall events	33.75 (±39.58)	28.42 (±37.05)	28.00 (±37.71)	76.60 (±46.25)	0	162	18.50
Total EMS fall events	96.09 (±108.65)	77.17 (±93.25)	85.33 (±103.92)	219.20 (±132.55)	0	386	56.50

In the 55 Tarrant County ZIP Codes examined, a total of 101 AMOB/VLL workshops were delivered between 2009 and 2011. Over this 3-year time period, 1,208 AMOB/VLL participants successfully met the criteria for completion of the intervention (i.e., attended five or more of the eight workshop sessions). Overall, 55.5% of the ZIP Codes offered one or more AMOB/VLL workshop in the 3-year period, with ZIP Codes offering an average of 1.84 (±2.71) workshops. The number of workshops offered within each ZIP Code ranged from 0 to 10, with 9.1% of ZIP Codes offering AMOB/VLL 6–10 times. The proportion of ZIP Codes that offered AMOB/VLL at least once remained consistent across the 3-year period, with 27.3% offering one or more workshops in 2009 (35 workshops), 32.7% in 2010 (34 workshops), and 34.5% in 2011 (32 workshops).

When comparing the average number of fall-related EMS calls by the number of AMOB/VLL workshops offered by ZIP Code, the average number of fall-related EMS calls was higher in ZIP Codes that offered more AMOB/VLL workshops.

**Figure 1:**
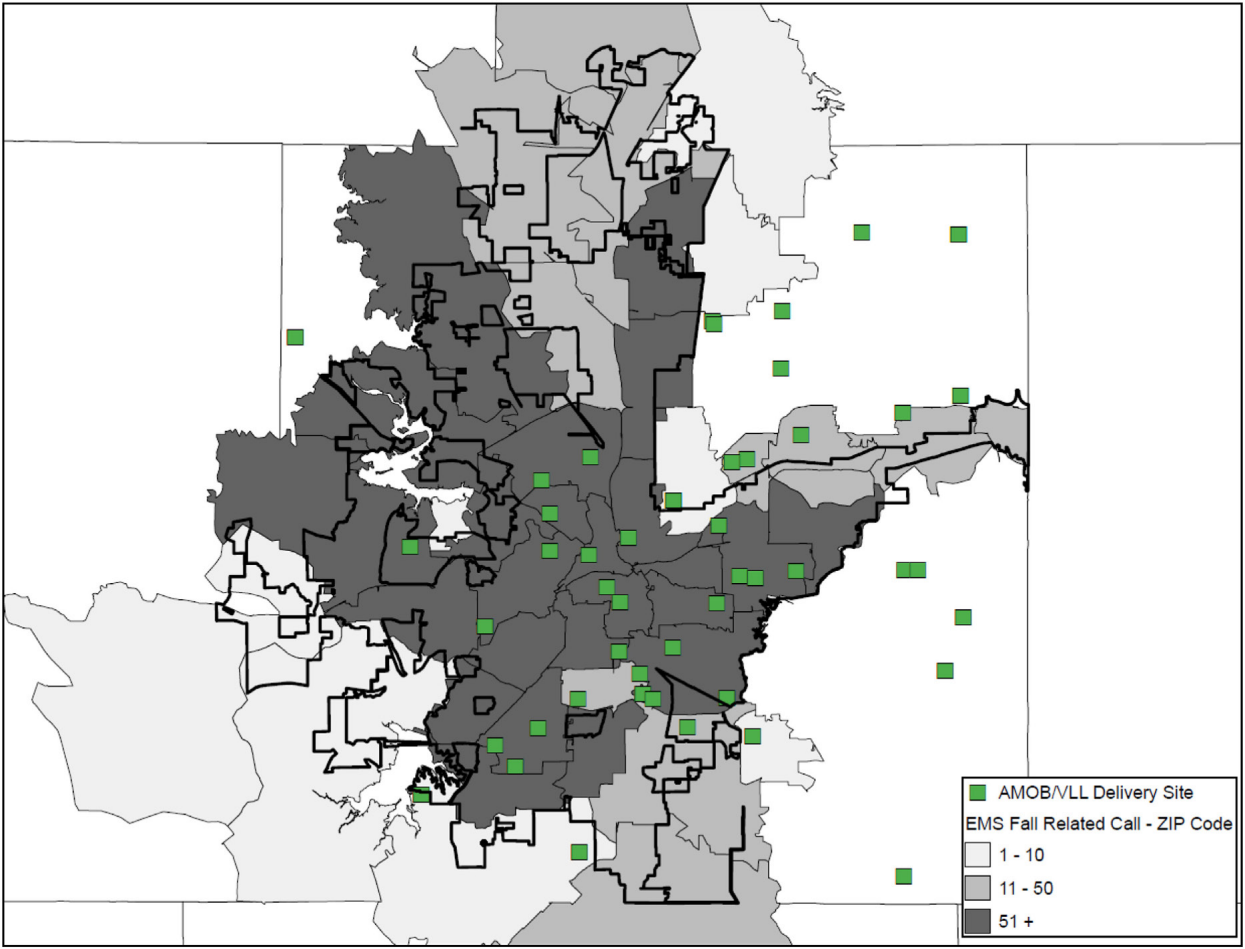
**A Matter of Balance/Volunteer Lay Leader Model (AMOB/VLL) delivery by ZIP Code based on the proportion of EMS Fall-related calls**.

A geographic information system was used to create a series of three maps illustrating the existence of at-risk areas in relation the availability of workshop delivery for asset mapping. Figure 1 identifies AMOB/VLL workshop delivery sites (identified as a single square) in relation to fall-related EMS calls aggregated at the ZIP Code and identified by the intensity (frequency) of calls represented by large shaded regions. Each ZIP Code is shaded based on the number or frequency of fall-related EMS calls received by the FWFD. Darker shaded areas indicate more fall-related EMS calls. As can be seen, a large proportion of AMOB/VLL workshops were delivered in ZIP Codes receiving 51 or more fall-related EMS calls. The overlap of several AMOB/VLL workshop delivery sites indicates successful reach to particularly at-risk areas with higher need (i.e., more relative EMS calls). Even so, many areas with 51 or more fall-related EMS calls were not served with an AMOB/VLL workshop (i.e., darker shaded areas without a single square). This map shows one approach to identifying community risk relative to assets as well as opportunities for service expansion.

**Figure 2:**
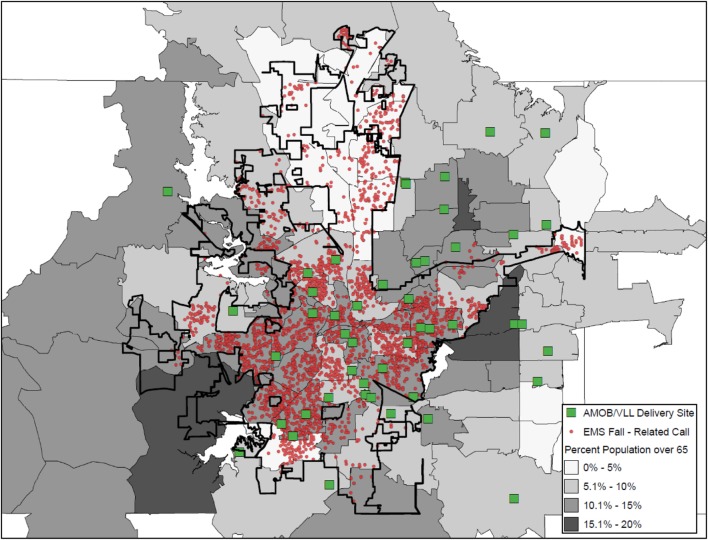
**A Matter of Balance/Volunteer Lay Leader Model (AMOB/VLL) delivery and EMS fall-related calls and ZIP Codes by proportion of residents age 65+**.

Figure 2 also illustrates the delivery of AMOB/VLL workshops (squares) in relation to fall-related EMS calls. In contrast to the shading approach in Figure 1 (i.e., shading ZIP Codes based on the frequency of fall-related EMS calls), Figure 2 presents the actual location of the EMS call was mapped (small circles). In Figure 2 shading is pulled from a separate data layer, now representing the percentage of residents aged 65 years and older, where the darker shaded areas indicate larger proportions of older adult residents. The vast majority of areas represented in Figure 2 had more than 5% of residents age 65 years and older as compared to 5% or less (no shading). As can be seen, a large proportion of fall-related EMS calls originated in ZIP Codes with 10.1–15% of the population being aged 65 years and older. Similarly, most of the AMOB/VLL workshops were delivered in areas with more than 5% of the population 65 years and older. Figure 2 also shows that the fall-related EMS calls (risk) were more concentrated in certain areas within individual ZIP Codes; whereas the shading in Figure 1 does not identify actual clusters (only aggregate numbers at the ZIP Code). Figure 2 also shows that areas with the largest concentration of older adult residents are not necessarily where AMOB/VLL workshops are delivered. This map shows more specified community risk relative to assets as well as more specified opportunities for service expansion.

**Figure 3:**
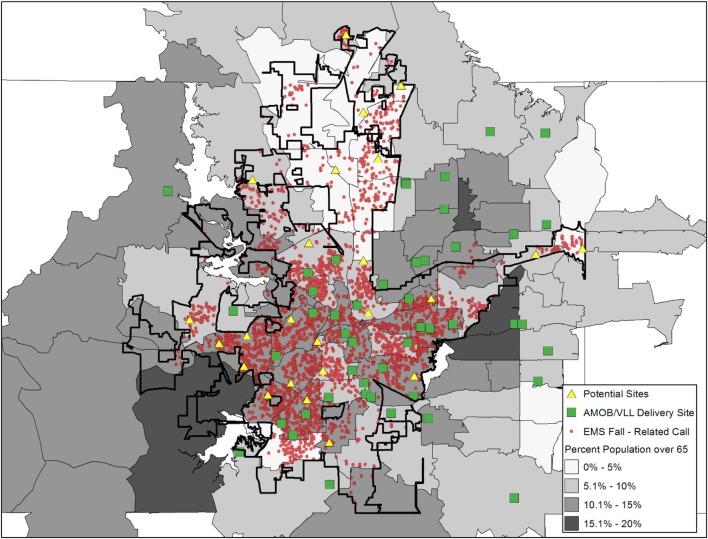
**A Matter of Balance/Volunteer Lay Leader Model (AMOB/VLL) delivery and EMS fall-related calls, ZIP Codes, and other resources by proportion of residents age 65+**.

Figure 3 is the same as Figure 2; however, an additional layer was added to show the location of agencies/organizations traditionally considered to be in the aging services network (e.g., senior centers, residential facilities, faith-based organizations). These agencies/organizations are depicted as triangles and represent potential partners who have not delivered AMOB/VLL. While this layer does not represent a full listing of agencies/organizations that could be recruited and engaged as delivery sites, this map shows specific details about organizations that can be targeted in high-risk areas for purposive service expansion.

## Discussion

Findings from this study show the utility of risk and asset mapping as related to fall-related risk and resources in community settings. Such approaches utilizing GIS have a range of benefits for strategic planning and mobilizing community action. Similar efforts can be carried out in multiple settings and with varied outcomes. Thus, this study may serve as a model in other approaches to asset mapping.

Findings from this study suggest that the study community was providing evidence-based fall prevention programming (AMOB/VLL workshops) in higher-risk areas, although many opportunities for service expansion were revealed. While offering community-based interventions like AMOB/VLL are important to serve high-risk areas, often these programs lack the ability to serve a large proportion of the aging population at risk for falling. For example, in communities where AMOB/VLL is embedded and regularly implemented, hundreds of older adults may be reached although thousands reside in the area. Therefore, efforts are needed to expand the training and delivery infrastructure to embed programs like AMOB/VLL throughout the community in a variety of locations (e.g., senior centers, faith-based organizations, health-care organizations, residential facilities) (29).

To complement these fall prevention efforts, findings from this study show that the identified risk areas can actually be opportunities for intervention. Stated another way, interventions can be delivered by first responders when responding to fall-related EMS calls. This intervention strategy holds great potential because EMS first responders are trusted members of the community with the ability to educate and influence health behavior (30). While some evidence-based fall prevention interventions delivered by emergency personnel exist (e.g., Remembering When) (31), other opportunities exist for EMS to adopt fall prevention efforts in their routine practice (32). Such interventions hold great potential to prevent falls because EMS first responders can educate older adults about fall-related risk, perform environmental scans to correct modifiable home safety issues, and make referrals to other fall prevention resources in the community. In addition to being effective, EMS-driven interventions can be cost-effective while not substantially increasing workload (32).

When interpreting the maps generated for the current study, it is important to consider the Ecological Fallacy as it applies to the concentration of the older adult population. In maps that present the distribution of AMOB/VLL delivery in relation to fall-related EMS calls and ZIP Codes by proportion of residents age 65 years and older, findings may initially appear counter-intuitive. One might expect that more falls would be reported in areas with the highest percentage of older residents; however, this was not always the case. Therefore, one must consider that the proportion of older adults in a given ZIP Code may not directly translate to the pure count of older adults in that ZIP Code relative to the total number of residents. In some cases, ZIP Codes may have a small number of total residents, thus the proportion of older adults seems large. Conversely, some ZIP Codes may have a large number of total residents, thus the proportion of older adults seems small. These aggregate proportions do not necessarily account for the total number of older adult residents (only the percentage relative to others aged 64 years and younger). Thus, care must be taken when interpreting the results based on aggregate data. It is always important to have an in-depth understanding of both the strengths and the limitations of the data presented to ensure accurate interpretations and clearly articulate service delivery and policy implications to your audience(s).

### Limitations

This descriptive study was not without limitations. First, data from 2009 to 2011 were used in this study and may not represent the most current rates of fall-related EMS data or AMOB/VLL workshop delivery. While the efforts to deliver evidence-based fall prevention programs in Tarrant County, Texas continue to progress, future studies should replicate these efforts with more recent data. Second, this study only examined one county, thus findings may not be widely generalizable. Future studies should replicate this effort in similar counties across the state or country or encompass larger service areas (e.g., entire states). That said, studies based on more localized sub-state boundaries may add additional context (e.g., available resources and stakeholder interest) to a given small area-specific analyses. Thus, public health departments or other agencies can develop similar projects focused on their own service areas. Third, risk mapping in this study was primarily classified by fall-related EMS calls and the proportion of ZIP Codes comprised of residents aged 65 years and older. It should be noted that including other factors can add richness to examine the multifaceted symptoms of falls risk at the environmental level. Examples of other risk factors that could be mapped include fall-related hospitalizations and fall-related emergency room visits. Similarly, locations of AMOB/VLL workshop delivery were the primary asset examined in this study. Including other factors can add richness to examine multilevel solutions available in communities. Examples of other evidence-based programs that could be mapped in communities include Stepping On, Tai Chi, and the Otago Exercise Program. Fourth, although fall-related EMS calls were examined in the current study, whether or not the older adult was transported or subsequently hospitalized is unknown. Further, fall-related injuries (if any) or outcomes associated with the fall event were not available.

### Recommendations

The below section contains recommendations for effectively using risk and asset mapping to enhance fall prevention efforts in communities. Utilizing these recommendations can assist decision makers to (1) assess community need and readiness for action; (2) evaluate the availability and accessibility of resources in a community; (3) identify service gaps; and (4) identify strategies to reach high-risk community members impacted by service gaps.

#### Select a Specific Population, Health Issue, and Data Sources

While there are numerous uses for data collected during an environmental scan of risks and assets, it is essential to narrow down a specific population and issue to be addressed by a particular initiative. Before taking up such an initiative, we recommend one determine what data are available prior to initiating this asset mapping process. This may also include reaching out to community resources (e.g., aging services sector organizations) or partnering with academic institutions to identify potential evaluation efforts. The purposive selection of data to be included in asset maps cannot be over emphasized. As such, this mapping process can be replicated multiple times for different populations (e.g., age groups, race/ethnicity) or health issues (e.g., falls, diabetes). Then, if justified by a theoretical relationship, maps can be combined for more comprehensive mapping and analyses.

#### Deliver the Message Efficiently without Overly Complex Maps

No matter how important a message is, message may be lost if it is not conveyed effectively. This is also true when displaying data *via* maps. For example, a good starting point in the mapping process for one organization may be identifying sociodemographic layers to see where the target population resides (e.g., population density, economic status, transportation systems). For other organizations, identifying hot spots for fall-related hospitalizations may be the starting point, again depending on the needs of that organization. Thus, a clear communication of the organization’s needs, mission, and intended outputs will likely dictate what data are presented. In any case, as seen in Figures 1–3, how the data are presented can affect the interpretation. Thus, limiting the number of layers or limiting the number of outcomes displayed in a single map may be needed in order to efficiently deliver your intended message. Collecting geographic layers containing information by definable borders (e.g., county, ZIP Code) and streets/highways can also enable successful linking of data across multiple disparate datasets. Thus, identifying an inventory of linkable layers and data can be a natural starting point when identifying which data one may be able to utilize. Sometimes generating a series of simple maps may be more informative than displaying overly complex maps with too many layers. In practice, limiting the data presented in a single map to only the most necessary information can help to avoid intensely complicated maps that may lose the intended message.

#### Identify Meaningful Community Assets

Because mapping is useful to identify risks and resources in communities, you must carefully identify organizations that may serve as opportunities to expand services. Highlighting all organizations in an area may be less informative if these organizations are incapable of delivering your intervention or service. For example, when thinking about the delivery of AMOB/VLL, we identified the delivery sites that offered the program in the 3-year period. Then, using the existing literature (16, 17, 20, 33), we identified other organizations that typically offer such evidence-based programs in the U.S. as potential partners and resources (e.g., senior centers, health-care organizations, residential facilities, faith-based organizations, tribal centers). Identifying key community partners and building strong relationships can help eliminate service gaps, reduce service duplication, and leverage limited community resources, which has implications for policy, practice, and cost.

#### Select the Most Appropriate Mapping Software

There are a variety of GIS and mapping software available for use. The functionality of these programs differs based on the field for which they were created. This also means that the data embedded within the programs (or those they have access to) also differ by discipline. Some programs are more expensive than others, thus understanding your organization’s ability to afford the program that best suits the need is important. Another important consideration is whether or not the program license includes technical assistance to help users best utilize the program. In some instances, this is an additional cost. Furthermore, it is important to consider whether or not someone in your organization has the skills to operate the program or the degree to which training is necessary (formal or informal). Examples of GIS and mapping software include: ArcGIS (used in the current analyses) and Tableau^1^; but many others exist. In addition to those identified here, free open-source GIS options can provide a free option for users with limited funding. Of note, identifying partners outside one’s organization may also be an option. For example, partnering with academic institutions with necessary expertise may be a viable option depending on mutual needs and resources.

#### There Are Several Ways to Approach Asset Mapping

However, as can be seen in Figures 1 and 2 of this study, the same data depicted differently (i.e., shaded ZIP Codes versus actual locations based on latitude/longitude coordinates) were capable of revealing higher-priority areas based on health risk and opportunities for service expansion. It is recommended that data with the most precision be acquired and incorporated into maps for increased specificity. This is especially so as the level of aggregation can vary dramatically as evidence by the fact that there are more than 70,000 Census Tracts (34) and nearly 40,000 ZIP Codes (35) within more than 3,000 counties (36) in the U.S. alone. Merging data can provide valuable insights, but we recommend reaching out to organizations or individuals with a working knowledge of the limitations of working with differing levels of geospatial aggregation. Thus, the level of geospatial aggregation can have serious implications when asset mapping. A basic visual display of potential geographic layers can be found at the U.S. Census Bureau’s website under their Geography Atlas.^2^ As stated before, this may require conversations with stakeholders and collaborators to understand the types of data available and the format in which they exist.

#### Engage Policy Makers and Other Stakeholders at Multiple Levels

Identifying and engaging key stakeholders throughout the planning process is critical to gain buy-in and ensure the evaluation efforts are in-line with mission of both individual organizations in the community but also a collective interests of multiple partners including local and state policy makers. This may be a critical step to ensure these key stakeholders are engaged in taking action on the identification of targets for outreach and other efforts, which are informed *via* the evaluation efforts (e.g., asset mapping). Being familiar with policy initiatives beyond one’s local community can be important when considering future funding from state or federal agencies, where appropriate. In addition, aligning evaluation efforts, in particular asset mapping efforts can provide valuable insight to key stakeholders given such mapping and evaluation efforts can more easily identify hot spots throughout a larger area (e.g., at the state level) (17). For example, the evaluation of fall-related hot spots (i.e., based on hospitalization discharge data) in relation to the delivery of AMOB/VLL has shown major gaps throughout Texas (17). Furthermore, asset mapping may lend itself to multiple mediums for effective dissemination. For example, AMOB/VLL delivery data collected *via* surveys from key stakeholders throughout Texas emphasizing resource allocation *via* mapping has been disseminated in the form of a policy brief (37). This and other tailored dissemination efforts may be needed depending on the intended audience (37). While these studies reflect what has been done relative to falls and hospitalization hotspots in Texas, other studies have used similar approaches to mapping disease prevention strategies. For example, one study (38) presented several examples of how public health departments could utilize GIS. Another example is where GIS was used to map hotspots for heart disease (i.e., areas with death rates higher than the national average) (39). Another example included mapping medical care infrastructure throughout the state of Minnesota (40). Yet, another example utilized mapping to assess the availability of stroke-related support groups relative to stroke-related hospital discharges (41). Many more applications can be gleaned from these examples and findings from the current study. A major takeaway is the ability to apply these skills to multiple projects, multiple locations, and diverse prevention efforts within public health and related disciplines.

#### Keep a History of Maps over Time

Maintaining an inventory of risk and asset maps is beneficial to identify trends. Organizations and communities that keep maps over a series of months/years are capable of identifying changes the prevalence of risk relative to service delivery, partner engagement, and persisting high-priority areas with resource gaps. Documenting the history of fall prevention efforts can demonstrate success over time, validate the continuation of community-based efforts, and justify decisions for ongoing and future funding.

#### Share Widely and Use as a Marketing and Leveraging Tool

Creating a community-wide dissemination plan of findings from risk and asset mapping activities has potential to promote successes among stakeholders to garner additional community support. Highlighting the risks and advancements in a certain area can stimulate the need for new partnerships and strengthen existing collaborations for fall prevention. Maps, findings, and recommendations should be disseminated using a variety of formats (e.g., websites, presentations, reports, social media, promotional flyers, publications) deemed appropriate for a variety of audiences (e.g., community-dwelling older adults, program participants, stakeholders, unengaged agencies/organizations, policy makers).

## Author Contributions

All authors contributed to the development and writing of this manuscript.

## Conflict of Interest Statement

The authors declare that the research was conducted in the absence of any commercial or financial relationships that could be construed as a potential conflict of interest.

## Footnotes

^1^http://www.tableau.com.

^2^https://www.census.gov/geo/reference/webatlas/.
